# The Impact of Facial Burns on Short- and Long-Term Quality of Life and Psychological Distress—A Prospective Matched Cohort Study

**DOI:** 10.3390/jcm12155057

**Published:** 2023-08-01

**Authors:** Alen Palackic, Camila Franco-Mesa, Inessa Beck, Steffen Nolte, Christian Tapking, Adriana C. Panayi, Annette Stolle, Valentin Haug, Christoph Hirche, Ulrich Kneser, Gabriel Hundeshagen

**Affiliations:** 1 Department of Hand, Plastic and Reconstructive Surgery, Burn Center, BG Trauma Center Ludwigshafen, 67071 Ludwigshafen am Rhein, Germany; alpalack@utmb.edu (A.P.); inessa.beck@hotmail.de (I.B.); mail@s-nolte.de (S.N.); christian.tapking@googlemail.com (C.T.); adriana.panayi@icloud.com (A.C.P.); annette.stolle@bgu-ludwigshafen.de (A.S.); vfm.haug@gmail.com (V.H.); christoph.hirche@bgu-frankfurt.de (C.H.); ulrich.kneser@bgu-ludwigshafen.de (U.K.); 2 Department of Surgery, University of Texas Medical Branch, Galveston, TX 77555, USA; camfranc@utmb.edu; 3 Department of Otorhinolaryngology, Head and Neck Surgery, Armed Forces Hospital Ulm, 89081 Ulm, Germany; 4 Department of Plastic, Hand and Reconstructive Microsurgery, Hand Trauma and Replantation Center (FESSH), BG Klinik Frankfurt am Main gGmbH, Academic Teaching Hospital of Goethe-University of Frankfurt, 60629 Frankfurt am Main, Germany

**Keywords:** burn injury, facial burn, quality of life

## Abstract

Burn injuries are a major healthcare challenge worldwide, with up to 50% of all minor burns located on the head and neck. With this study, we sought to describe the effect of facial burns (FB) on health-related quality of life through a prospective and matched cohort study design. Patients completed the 36 Item Short Form (SF-36) and the Hospital Anxiety and Depression Scale (HADS). Results were analyzed based on the distribution of datasets. In total, 55 patients with FB and 55 age-and sex-matched candidates were recruited. The most common mechanism of thermal injury was burns from flames. The FB group scored lower in physical and psychological dimensions than the control group, both acutely and one year after injury. An analysis of each domain showed that subjects in the FB group trended toward improvements in their score after one-year post-burn in physical functioning (acute: 71.0 ± 29.2; one-year: 83.7 ± 23.9; *p* = 0.02) and bodily pain (acute: 58.5 ± 30.3; one-year: 77.9 ± 30.5; *p* = 0.01) domains. Additionally, the FB group had significanlyt higher scores for anxiety (FB: 4.8 ± 3.2; control: 2.5 ± 2.8; *p* = <0.002) and depression (FB: 3.9 ± 3.5; control: 2.1 ± 2.7; *p* = 0.01) compared to the control. In conclusion, facial burns are associated with physical and psychosocial deficits, as well as elevated levels of psychological distress.

## 1. Introduction

Adequate treatment and the reconstruction of burn injuries is a major healthcare challenge, with the global annual incidence rate of burns reaching up to 7 to 12 million cases [1]. The head and neck region is a commonly affected area, with 50% of all minor burns located in this region, and at least half of patients with major burns have some degree of facial involvement [2]. More so than other parts of the body, the facial area plays functional and aesthetic roles that are crucial for self-concept and social interaction [3,4]. The delicate and complex arrangement of neurovascular bundles, subcutaneous tissue, and osseous protuberances found in the head and neck present multiple challenges when treating a burn injury [2,3]. Additionally, the hypermetabolic and catabolic response to thermal lesions creates a dysregulated hyperinflammatory state [5,6]. As a result, patients suffer from severe scarring processes that are associated with facial disfigurement and long-term disability [3,7]. The lack of self-recognition, acceptance, and/or coping mechanisms coupled with facial motor or sensory impairments can trigger a series of psychological changes that impact the quality of life (QOL) [8].

According to the World Health Organization, quality of life (QOL) is “an individual’s perception of their position in life in the context of the culture and value systems in which they live and concerning their goals, expectations and standards”. This includes health-related QOL (HRQOL), that is, the individual’s perceived mental and physical health. Chronic and debilitating conditions such as burn injuries are known to have detrimental effects on HRQOL [9]. While multiple factors such as the severity of the wound, socio-economic status, coping mechanisms, personality, the number of interventions, and impaired functionality have been associated with decreased HRQOL, the localization of the injury to the face is a notably identified factor that affects perceived health outcomes [3,8]. Although a correlation between facial burns and disability is well-recognized, details regarding its effect on HRQOL are scant [3]. Given that data available on the topic are scarce [10], the purpose of this study is to describe the effect of facial burns on HRQOL through a prospective and matched cohort study design carried out in a level one burn center. 

## 2. Materials and Methods

A prospective observational matched controlled study was conducted at a single, level one burn center in Germany. Patients who were admitted to the hospital between March 2019 and August 2020 with partial or full-thickness facial burns (FB) were above the age of 14, provided informed consent, and were enrolled in the study. Eligible burn etiology for inclusion in the study included fire/flame, electrical, scald, and contact burns. All other burn injuries which did not meet these criteria were ineligible for this study. In addition to baseline clinical data, including the total body surface area (TBSA) burned, the extent of facial burns was quantified as the burned area in percent of the total facial area in each patient. Patients were evaluated seven days prior to their planned discharge to mitigate the hyperacute effects of the burn as a confounder. A representative healthy control cohort was matched 1:1 based on age and sex and is included in the study (CTR). The patient collective of this current study has been previously investigated in a separate, unrelated study by our group. A detailed description and the data of this study have been previously published [11]. 

The data were extracted in Excel using a predetermined and password-protected form of data collection. All patients responded to the following two surveys: a 36-Item Short Form (SF-36) and the Hospital Anxiety and Depression Scale (HADS). The control group completed the forms once and the FB group twice, once before discharge and once at one-year post-injury. 

Briefly,

- The 36-Item Short Form (SF-36) is a widely employed tool that is used to evaluate QOL [12]. Thirty-six of the items are grouped to form eight domain subscales: PF: physical functioning, RP: physical role functioning, BP: bodily pain, GH: general health perception, VT: vitality, SF: social role functioning, RE: emotional role functioning, MH: mental health, in an aim to provide a measure of overall well-being: [12]. Despite its ease of use and application, this tool meets rigorous standards to provide validity and reliability in multiple medical settings [13,14].
- The Hospital Anxiety and Depression Scale (HADS) is a fourteen-question instrument that is used to measure psychological distress in different clinical settings, with an emphasis on anxiety and depression [15]. Each item is ranked on a 4-point scale (0–3) based on severity, where 0 equals no symptoms, and 3 equals severe symptoms. Scores for each subscale can range from 0 to 21 with scores categorized as follows: 0–7 (normal), 8–10 (mild), 11–14 (moderate) 15–21 (severe). A higher score indicates more depressive symptoms [16]. Unlike other tools, HADS can change during the course of the disease allowing for the identification of psychological changes associated with treatments or interventions [15].

For the general QoL assessment, we performed detailed analyses of the two main constructs of the SF-36 score—physical (PSC) and psychosocial (MSC) components—as well as of each SF-36 domain to determine differences between the FB and CTR groups before discharge and one-year post-burn. Following the initial analysis of both cohorts, a subgroup analysis was performed on the results of SF-36 in patients who had inhalation trauma on admission (II) and those who were classified as having a “complicated” FB (COMP), defined by the presence of at least two of the following: TBSA > 20% burned, need for surgery to the face, or visible scars on the face at 1-year post-burn. 

### Statistical Analysis

Continuous outcome parameters were compared using Student’s unpaired *t*-test, Mann–Whitney U test, or one-way ANOVA, depending on the normal distribution of datasets assessed by the Shapiro–Wilk test. Paired repeated nonparametric measurements were analyzed with the Wilcoxon matched-pairs signed rank test. Contingencies were analyzed using Fisher’s exact test. Statistical analyses were performed using GraphPad Prism version 7.00 for Windows (GraphPad Software, La Jolla, CA, USA). The results are presented as the mean ± standard deviation (SD), or medians, inter-quartile ranges (IQR), and ranges according to the normality of data distribution. Statistical significance was accepted at *p* < 0.05.

## 3. Results

### 3.1. Patient Population

In total, 73 patients were potentially eligible for inclusion, of which 55 patients with FB consented to be enrolled in the study (participation rate: 75%) and completed the SF-36 and HADS surveys. Fifty-five age- and sex-matched volunteers were recruited and matched as controls (CTR). Thirty patients (55%) completed the surveys at one-year post-burn (lost-to-follow up 45%). Both groups were comparable in age, sex distribution, and smoking status. Demographic data of FB and CTR patients are presented in Table 1. 

The median age at injury was 37 years (IQR: 24 years). The most common mechanism of thermal injury was a fire/flame burn (85%), followed by an electrical burn (7%), scald burn (6%), and contact (2%). The median burn TBSA was 11% (IQR: 29%), with an average involvement of 50% of the face (IQR: 40%). Most facial burns were treated without any surgical interventions and healed without visible scarring at one-year post-burn. Nine patients (16%) required surgery to the face and upper neck in the acute setting of a hospital stay, whereas nine had visible scars, and four patients (7%) required additional reconstructive procedures within one year after injury. Full details of the FB characteristics are provided in Table 2.

### 3.2. Physical and Psychosocial Functioning Summary Scores

**Figure 1:**
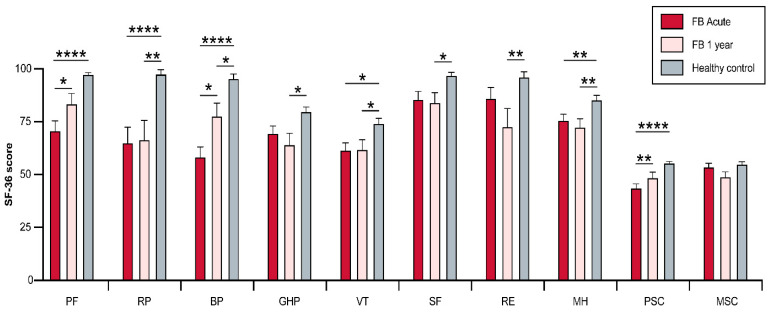
Short (acute) and long-term (one year) assessment of SF-36 scores after facial burn. Visualization of the analysis of FB patient scores versus a healthy cohort. FB, facial burn; PF, physical functioning; RP, physical role functioning; BP, bodily pain; GH, general health perception; VT, vitality; SF, social role functioning; RE, emotional role functioning; MH: mental health; PSC, physical summary scale; MSC, mental summary scale; * <0.5, ** <0.01, **** <0.0001.

Overall, subjects in the FB group scored lower in the physical and psychological dimensions than the CTR group, both acutely and one year after injury (Figure 1). The CTR group had significantly higher score in physical functioning summary scores (PSC) than the burn-injured participants in the acute phase (FB: 43.9 ± 10.6; CTR: 55.5 ± 3.5; *p* ≤ 0.0001), and there was no significant difference at the one-year post-burn time-point (FB: 48.8 ± 12.0; CTR: 55.5 ± 3.5; *p* = 0.06). Subjects in the FB group trended toward a significant improvement between the acute and one-year post-burn (acute: 43.9 ± 10.6; one-year: 48.8; *p* = 0.004). There was no significant difference in the mental summary score (MSC) between both groups acutely (FB: 53.8 ± 9.4; CTR: 55.2 ± 6.5; *p* > 0.9) and one-year post-burn (FB: 49.2 ± 10.8; CTR: 55.2 ± 6.5; *p* = 0.053; Figure 1). There was no significant declining trend in the FB group from the acute to the one-year post-burn time-point (acute: 53.8 ± 9.4; one-year: 49.2 ± 10.8; *p* = 0.1). 

### 3.3. Physical and Psychosocial Domains

Detailed analysis of each domain showed that subjects in the FB group displayed significant improvements one-year post-burn in the PF (Acute: 71.0 ± 29.2; One-year: 83.7 ± 23.9; *p* = 0.02) and BP (Acute: 58.5 ± 30.3; One-year: 77.9 ± 30.5; *p* = 0.01) domains (Figure 1), while no significant differences in the scores of the other six domains (RP, SF, MH, RE, GH, VT) were found. Acutely, subjects in the CTR group scored significantly higher in the PF (FB: 71.0 ± 29.2; CTR: 97.6 ± 4.5; *p* ≤ 0.001), RP (FB: 65.2 ± 45.4; CTR: 97.8 ± 13.8; *p* ≤ 0.001), BP (FB: 58.5 ± 30.3; CTR: 95.7 ± 14.0; *p* ≤ 0.001), MH (FB: 75.8 ± 17.7; CTR: 85.6 ± 13.7; *p* = 0.005) and VT (FB: 61.7 ± 21.4; CTR: 74.3 ± 16.6; *p* = 0.015) dimensions than subjects in the FB group. No differences were noted for the other three dimensions (SF, RE, GH; Figure 1). At one-year post-burn, the healthy control scored significantly higher in all eight domains compared to the FB group. 

### 3.4. Complicated Facial Burns (COMP)

**Figure 2:**
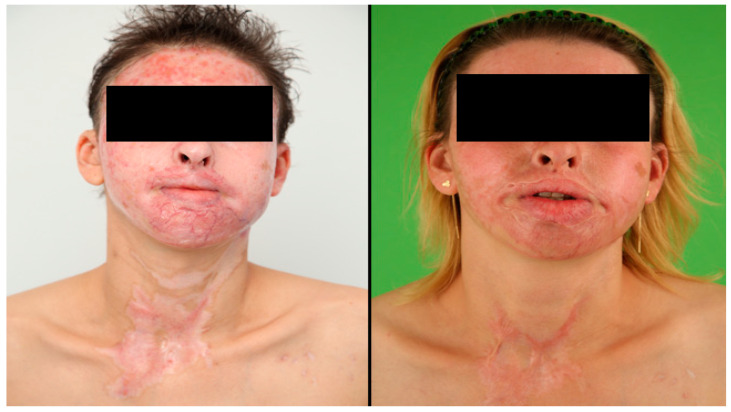
Short and long-term (one-year) results after severe burn injury with facial involvement. This patient suffered from a severe burn injury with the development of hypertrophic scarring in the face and neck area.

Further sub-group analysis was performed to determine differences in the SF-36 scores between complicated (COMP) and uncomplicated burns, acutely and one-year post-burn. Overall, at the acute time-point, the COMP group tended to have lower scores than uncomplicated burns, with the greatest difference in the RE domain, although no significance could be established (Supplementary Materials). Comparable scores in PCS were noted between the two groups in the acute phase (COMP: 41.1 ± 21.7; FB: 44.6 ± 10.23; *p* > 0.9). There was also no significant difference in MCS between the groups both acutely (COMP: 49.2 ± 10; FB: 55.4 ± 8.7; *p* = 0.19) and at one-year post-burn (COMP: 47.6 ± 11.7; FB: 49.7 ± 10.7; *p* ≥ 0.9). The MSC score in the COMP group displayed a declining trend from the acute to the one-year post-burn time-point, although no significance was found (acute: 49.2 ± 10; one year: 47.6 ± 11.7; *p* > 0.9). Detailed analysis of each domain showed that patients in the COMP group scored, on average, lower than uncomplicated burns in all eight domains in the acute phase and one-year post-burn. The largest difference was in the RE dimension, again with no statistical significance (Acute, COMP: 66.7 ± 44.1; FB: 90.5 ± 26.3; *p* = 0.42; One-year, COMP: 61.9 ± 48.8; FB: 76.7 ± 4.2; *p* > 0.9) (Figure 2).

### 3.5. Psychological Distress (HADS)

**Figure 3:**
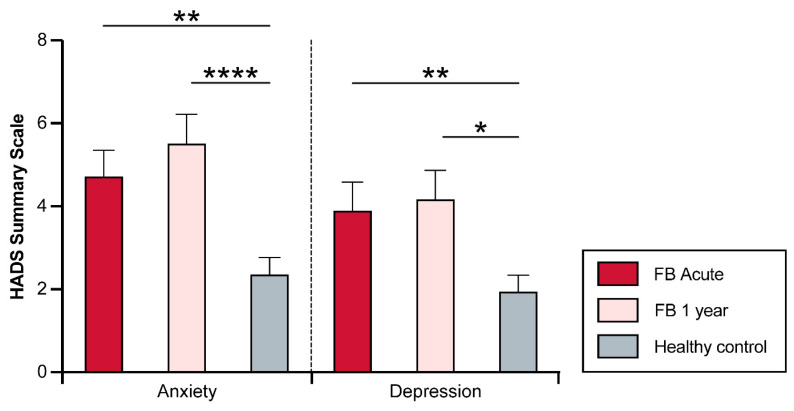
Short (acute) and long-term (one year) assessment of HADS scores after facial burn. Visualization of the scores in facial burn patients versus healthy cohort. HADS, Hospital Anxiety and Depression Scale; FB, facial burn; ns, not significant; * <0.5, ** <0.01, **** <0.0001.

No significant increase in the HADS scores was found one year after injury (Anxiety, acute: 4.8 ± 3.2; Anxiety, one-year: 5.6 ± 3.6; *p* ≥ 0.9; depression, acute: 3.9 ± 3.5; Depression, one-year: 4.2 ± 3.6; *p* > 0.9). In the acute setting, compared to the CTR group, subjects in the FB group had significantly higher scores for anxiety (FB: 4.8 ± 3.2; CTR: 2.5 ± 2.8; *p* < 0.002) and depression (FB: 3.9 ± 3.5; CTR: 2.1 ± 2.7; *p* = 0.01). One year after the burn injury, subjects in the FB group had significantly higher scores of anxiety (FB: 5.6 ± 3.6; CTR: 2.5 ± 2.8; *p* < 0.001) and depression (FB: 4.2 ± 3.6; CTR: 2.1 ± 2.7; *p* = 0.005) than the CTR group (Figure 3). 

## 4. Discussion

To the best of our knowledge, this is the first prospective matched cohort study to investigate the short- and long-term impact of common facial burns on various components of QOL and psychological distress. The findings of our study could help to provide a better understanding of the psychosocial burden of patients with facial burns and its short and long-term impact on self-perceived QOL and psychological distress. 

This current study shows that common facial burns are associated with physical and psychosocial deficits in the subacute phase of injury and persist after a year of injury. Furthermore, our analysis suggests that the well-being of common FB patients tends to improve over time; however, despite this improvement, levels on all physical and mental health status sub-scales remain lower compared to normative values. In particular, one year after injury, all measured physical and mental subscales were significantly lower than those in the average population. Furthermore, we identified that patients who were severely burned, received surgery to the face, or had visible scars one year after injury and, hence, were classified as having complicated burns tended to have worse self-perceived physical and psychosocial outcomes compared to uncomplicated burns. 

Several studies have investigated the negative impact of facial burns on self-perceived QOL [17,18]. In contrast to our investigation, most studies have focused on the impact of facial burn scars rather than common burns of various degrees [19,20,21]. Our statistical attempt to distinguish a population at risk with “complicated” rather than “uncomplicated” facial burns could influence clinical guidance. Although a trend of worse outcomes for complicated compared to uncomplicated facial burns was noted, our findings did not reach statistical significance. These findings suggest that the severity of a burn and the presence of visible scars do not necessarily correspond with significantly worse self-perceived physical and psychosocial outcomes compared with uncomplicated burns. The potential reasons for these findings remain speculative: the location of the injury to the face is one of the notable identified factors that affect perceived health outcomes [3,8]. As suggested by other studies, even minor injuries to the face can have a substantial negative short- and long-term psychological impact on patients [22]. Similar to burn victims with pathological scars, patients with uncomplicated burn injuries may develop a negative self-image causing challenges with social collaboration and the stigma attached to their physical appearance [20].

We observed a trend toward worse self-perceived physical and psychosocial measures in complicated burns compared to uncomplicated. Our findings are, to a degree, in agreement with other studies that emphasize the physical and psychosocial deficits of complex facial burns [21]. A study by Gandolfi et al., for example, described patients with significantly lower SF-36 scores in several sub-scales [21]. Although our methodology and follow-up time-points differ, our findings support this study in the aspect that complicated facial burns are associated with below-average scores in mental constructs of HRQOL measures. Hence, it is essential to recognize the need for early attention, long-term follow-up, and the empathic understanding of subjective limitations in QOL for every facial burn patient. Clinicians can implement these surveys in daily rounds or follow-up visits to plan early and promptly adjust multimodal patient-centered treatments, such as psychological treatments, social help, and surgical interventions. 

Self-perceived HRQOL is a frequently studied parameter in burn patients [23,24,25,26,27]; however, data on facial burns are sparse. For this present study, we administered the SF 36 survey, a commonly employed tool in the field of burn care that evaluates physical and psychosocial well-being, and our findings have added new insights to the literature. Other surveys, such as the Perceived Stigmatization Questionnaire (PSQ), have been implemented for investigating self-perceived HRQOL in patients with facial disfigurement [19,20]. This was not in the scope of this present study; however, our collective of complicated burns experiencing visible scars scored worse than uncomplicated burns in several physical and mental subscales, especially in the emotional role subscale. The reasons for these findings are speculative; studies have shown that perceived stigma due to scarring is associated with negative emotions and psychological burdens [28]. The PSQ has been successfully implemented in the field of burns, and future studies should expand upon our findings and investigate the grade of perceived stigmatization in patients of a larger cohort and especially those with severe facial burns. 

In our second analysis, we investigated the level of anxiety and depression in facial burn patients. Our findings show that patients with facial burns have significantly elevated levels of anxiety and depressive symptoms, both acutely and at the one-year time-point after the injury, compared to healthy volunteers. The current body of literature has reached no consensus on this topic, with the existence of debates on whether depressive symptoms play a significant role after facial burn injury. A study by Williams et al. suggested that the visibility of the burn was a useful predictor of psychological outcomes [29]. A study by Thombs et al., in contrast, found no significant direct correlation between facial burns and symptoms of depression. However, the authors of this study also identified that mild symptoms of depression were present in 46% of a patient cohort who had sought consultation in a burn reconstruction clinic [30]. Although this study utilized a different assessment instrument, the findings of our study are in line with the results of Thombs’ study, as the average and most of our study collective did not reach a score over seven, suggesting low to no depressive symptoms after facial burns. Nonetheless, our findings also identified significantly elevated anxiety and depression levels compared to those of healthy controls, and therefore, clinicians should monitor for anxiety and depressive symptoms. 

The limitations of this study should be acknowledged. Due to its single-center pilot study design, no strong conclusions can be drawn. The overall cohort size was small, and the results, despite showing statistical significance, should be interpreted with caution. Future studies should expand upon our findings with larger patient collectives with a focus on cohorts that have more severe facial burns. Our study utilized a 12-month final follow-up time-point, which might not reflect the final stage of recovery. Further research should include later follow-up time-points to investigate the QOL outcomes in this special patient population. There have been multiple validation studies of the SF-36 survey, including in different languages; to the best of our knowledge, however, it has not yet been specifically validated in a German-speaking collective of patients with facial burns [24,31]. Furthermore, this study is a secondary analysis of a prospectively collected patient sample [11]. A power calculation was based on this prior study’s endpoint, resulting in the inclusion of 55 patients. Due to the analysis of a secondary endpoint of that same study, no separate power analysis was performed for the questionnaire endpoints reported in this manuscript. Finally, pre-existing psychiatric morbidities, which have been shown to be a major factor affecting the QOL in burn patients, were not considered [32]. Despite these limitations, our findings provide insights into the patterns of recovery that may help us in improving the QOL and psychological distress in patients with facial burns. 

## 5. Conclusions

Although the appearance and return to function for the majority of burns improve over time, in this study, we found that even at one-year post-injury, the measured QOL outcomes were significantly lower than those of healthy controls. Complicated facial burns tended to have lower scores than uncomplicated ones, especially in the psychosocial dimensions of the SF-36 survey. Facial burn patients are seen to experience significantly elevated anxiety and depression levels compared to healthy controls; these challenges were seen to persist at the one-year post-burn time-point. Future studies should expand on our findings with larger patient cohorts placing particular focus on patients with more severe facial burns.

## Figures and Tables

**Table 1:** Baseline demographics of patients and control subjects.

	Group FB (*n* = 55)	Group CTR (*n* = 55)	*p* Value
Age; Median (IQR)	37.0 (24)	38.5 (26)	0.69
Sex; *n* (% male)	47 (85)	47 (85)	0.99
Smoker; *n* (%)	21 (39)	12 (22)	0.09

**Table 2:** Baseline characteristics of the FB group. Reported as *n* (%), unless otherwise stated.

Characteristic	Group FB (*n* = 55)
TBSA; median (IQR)	11 (29)
Facial involvement (% of facial area)	50 (40)
Cause of burn	47 (85)
Flame/Flash	4 (7)
Electricity	3 (6)
Steam	1 (2)
Contact	50 (40)
Inhalation trauma	4 (7)
LOS; median (IQR)	17 (25)
LOS ICU; median (IQR)	3 (18)
LOS/% TBSA; median (IQR)	1.5 (1.3)
Mechanical ventilation; median days (IQR)	0 (1)
Mortality	0 (0)
Need for surgical treatment	
Acute	9 (16)
Within one year	4 (7)

TBSA, total body surface area; LOS, length of stay; ICU, Intensive Care Unit; IQR, interquartile range.

## Data Availability

The data presented in this study are available on request from the corresponding author. The data are not publicly available due to the sensible nature of patient-derived information.

## Supplementary Materials

The following supporting information can be downloaded at: https://www.mdpi.com/article/10.3390/jcm12155057/s1, Figure S1: Short (acute) and long-term (one year) assessment of SF-36 scores after facial burn. Comparison of uncomplicated versus complicated facial burns.
