# Supplementary material for: The Impact of Facial Burns on Short- and Long-Term Quality of Life and Psychological Distress—A Prospective Matched Cohort Study
Source: J Clin Med. 2023 Aug 1;12(15):5057. doi: 10.3390/jcm12155057 (PMC10419582; doi:10.3390/jcm12155057)
Supplement: Supplementary file 1 [file jcm-12-05057-s001.zip › jcm-2471708-supplementary.pdf]

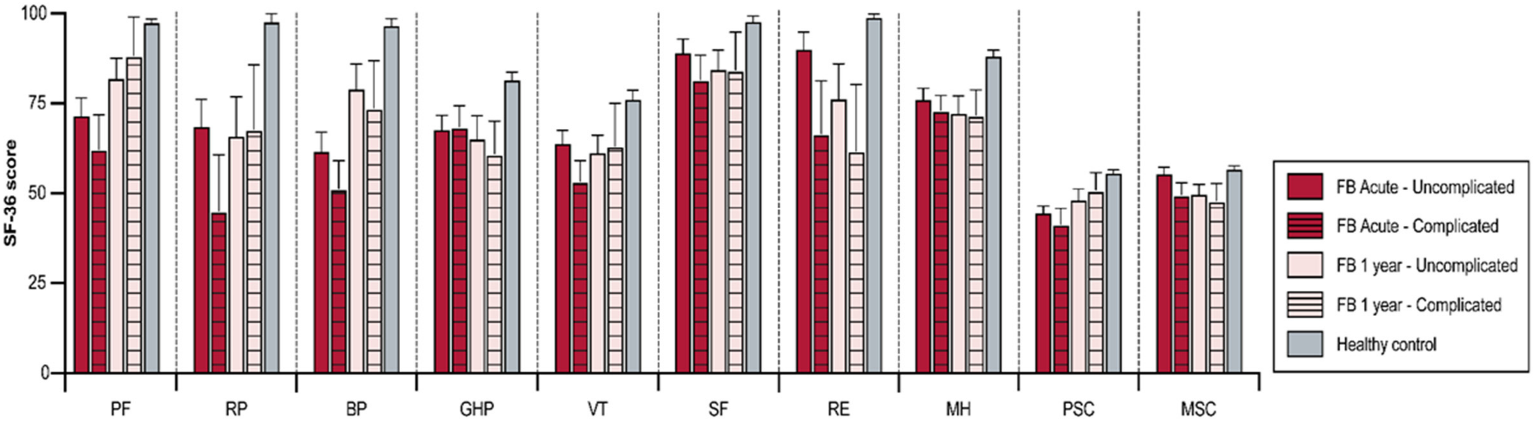

Figure S1. Short (acute) and long-term (one year) assessment of SF-36 scores after facial burn. Comparison of uncomplicated versus complicated facial burns. FB, facial burn; complicated FB, complicated facial burn with presence of at least two of the following: TBSA > 20% burned, need for surgery to the face, or visible scars on the face at one-year post-burn; PF, physical functioning; RP, physical role functioning; BP, bodily pain; GH, general health perception; VT, vitality; SF, social role functioning; RE, emotional role functioning; MH: mental health.
